# Modelling to inform next-generation medical interventions for malaria prevention and treatment

**DOI:** 10.1038/s43856-023-00274-0

**Published:** 2023-03-25

**Authors:** Narimane Nekkab, Josephine Malinga, Lydia Braunack-Mayer, Sherrie L. Kelly, R. Scott Miller, Melissa A. Penny

**Affiliations:** 1 Swiss Tropical and Public Health Institute, Allschwil, Switzerland; 2 University of Basel, Basel, Switzerland; 3 Gates Medical Research Institute, Cambridge, USA

**Keywords:** Malaria, Infectious diseases, Computational biology and bioinformatics

## Abstract

Global progress against malaria has stagnated and novel medical interventions to prevent malaria are needed to fill gaps in existing tools and improve protection against infection and disease. Candidate selection for next-generation interventions should be supported by the best available evidence. Target product profiles and preferred product characteristics play a key role in setting selection criteria requirements and early endorsement by health authorities. While clinical evidence and expert opinion often inform product development decisions, integrating modelling evidence early and iteratively into this process provides an opportunity to link product characteristics with expected public health outcomes. Population models of malaria transmission can provide a better understanding of which, and at what magnitude, key intervention characteristics drive public health impact, and provide quantitative evidence to support selection of use-cases, transmission settings, and deployment strategies. We describe how modelling evidence can guide and accelerate development of new malaria vaccines, monoclonal antibodies, and chemoprevention.

## Abstract (web summary)

Nekkab, Malinga, Braunack-Mayer et al. discuss how modelling can be incorporated early on in the research and development of malaria tools alongside clinical evidence and expert opinion. In addition, population models can provide estimates of potential effectiveness of novel interventions to inform product criteria and support decision-making.

## Introduction

There is an urgent need to accelerate development of novel malaria interventions to prevent infections and severe disease. Progress to reduce the impact of malaria has stalled worldwide alongside disruptions to services during the COVID-19 pandemic. Malaria cases in the African Region increased from 225 per 1000 population at-risk in 2019 to 234 in 2020 and remained similar at 229 cases per 1000 population in 2021^1,2^. Estimated malaria deaths in this region rose from 568,000 in 2019 to 625,000 in 2020 and declined marginally to 619,000 deaths in 2021. Moreover, the emergence of partial resistance to treatment with artemisinin in some parts of Africa threatens the efficacy of artemisinin-based combination therapies. Increasing insecticide resistance and invasion of the primary mosquito vector in India, *Anopheles stephensi*, into Africa may also lead to increasing transmission in urban areas, which could reverse progress made over recent decades^3^.

While the 2021 World Health Organization’s (WHO) recommendation of the first malaria vaccine, RTS,S, was a major milestone, the vaccine took 30 years to develop, test, and pilot, and its availability, acceptance and use has not yet been demonstrated^4^. Furthermore, current malaria prevention measures do not reach all children. For example, seasonal malaria chemoprevention (SMC) is only recommended and deployed in highly seasonal settings with low drug resistance risk. Implementation of intermittent preventive treatment of malaria in pregnancy using sulfadoxine-pyrimethamine (IPTp-SP) is not recommended in low transmission or high SP resistance settings and there is limited guidance on the adverse consequences of drug–drug interactions^5^. Resource constraints, limited access to care, and low chemoprevention adherence in children and pregnant women, who are vulnerable to severe outcomes, continue to be major challenges. The development of novel interventions for malaria prevention must be accelerated to meet current and future needs. Incorporating all forms of evidence to guide decision-making early on during the product development pipeline will be vital in accelerating the process.

Global funders, regulatory agencies, and researchers are expanding their pipelines for next-generation medical interventions for malaria prevention, guided by reference documents for candidate selection and investment decision-making in research and development (R&D). While guidance documents vary, they essentially outline the necessary characteristics required to support development decisions and optimisation of innovative products. The WHO developed Preferred Product Characteristics (PPCs), informed by technical working groups and public consultation, to guide and promote the development of various malaria prevention interventions such as vaccines, chemoprevention, and, recently, for monoclonal antibodies (mAbs) as well as vector control interventions. Medicines for Malaria Venture (MMV) also uses Target Candidate Profiles (TCPs) to support product development partnerships, pipeline development for long-acting injectable drugs, and repurposing, recombining, and developing antimalarials for SMC^6^. The Bill & Melinda Gates Foundation is developing intervention Target Product Profiles (iTPPs) to optimise investment in three next-generation medical interventions for prevention: novel immunological seasonal therapeutics using mAbs and long-acting injectable drugs, multi-seasonal interventions using second-generation malaria vaccines, and novel candidates or repurposed drugs for second-generation oral chemoprevention. In the case of malaria iTPPs for example, the documents define characteristics for development of novel intervention by how they are likely to be used and their indication, product components, target population and setting, safety, formulation, and pre-qualification date targets among other criteria for a base and upside case.

Prioritisation should be given to establishing a comprehensive evidence base to iteratively inform guidance documents and support selection of the most promising candidates, thus accelerating the development of novel medical interventions to prevent malaria. We argue that this evidence base should encompass the full range of quantitative and qualitative findings from clinical trials, modelling, and expert opinion. In the case of malaria prevention, selecting candidates that maximise the potential to achieve high public health impact and accelerate implementation requires decisions based on well-informed product criteria accounting for potential R&D bottlenecks, resources, and timeframes. Importantly, modelling evidence should be incorporated early in the drug development process, as modelling can uniquely link product criteria or properties to likely public health impact, which is not possible before or during clinical trials and is only possible after implementation. In addition to modelling existing interventions, modelling can estimate the potential range of impact on disease burden of a novel intervention to support building a value proposition document used to attract commercial development partners and engage with diverse stakeholders and partners that include those in malaria country programs and health agencies. Such modelling analyses also allow translation of clinical trial evidence to population impact across transmission settings, deployment strategies, or use-cases. Here, we focus our discussion on malaria prevention tools; however, this framework can also be expanded to malaria treatment, vector control, and to other diseases.

## Establishing an iterative approach and framework to generate a comprehensive evidence-base

**Fig. 1:**
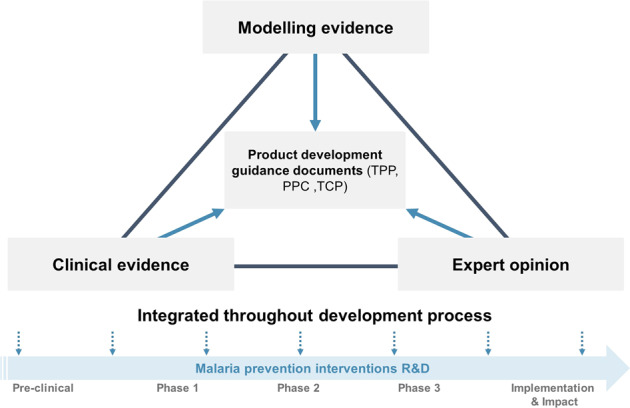
Iterative evidence generation process to develop guidance and accelerate development. An iterative and collaborative approach between clinical investigators, modellers, and malaria experts to generate evidence to continuously inform guidance documents. Modelling provides evidence on trade-offs between intervention characteristics and minimum criteria informed by predicted public health impact and supported by clinical evidence to define parameter values and generating evidence for priority scenarios defined by experts for different use-cases. Clinical studies provide initial efficacy evidence of first candidates and with improved design and planning informed by modelling evidence to support key evidence for selection criteria of future candidates. Through stakeholder engagement, expert opinions provide well-informed ranking of priorities for the evidence generation process including identifying scenarios to model for different use-cases and intervention feasibility for implementation.

Anecdotally, R&D for malaria interventions seems to take longer than for other diseases. This is due, in part, to higher investment risk since drug and intervention combinations are often needed to demonstrate sufficient effectiveness, on account of malaria’s varied and changing epidemiological landscape. In addition, lack of a dual market dominated by the public sector in malaria endemic settings which are often low and middle-income countries provides little to no financial return as opposed to the travellers market which can be more lucrative^6,7^. While increased global investment has helped accelerate development in recent years, candidate selection guidance documents and priority use-cases are mainly informed by expert opinion and results of early stage clinical trials. Guidance documents have an important role to play in shortening R&D timelines and reducing costs, targeting priority use-cases and unmet needs, while ensuring selected candidates have a higher probability of demonstrating high impact for their given use-case. We posit that well informed decision-making along the product development pipeline, from discovery, proof-of-concept, and program implementation to impact, requires the iterative use of a comprehensive evidence-base that includes modelling evidence and allows for decisions to be adapted as new evidence becomes available (Fig. 1).

Modelling can inform different priority health targets, as the models can consider both the patient-level benefit of novel prevention products (i.e. the individual-level protection it provides) as well as the population-level benefit of deploying such a product to communities by quantifying the additional public health gains. Pharmaceutical approaches have traditionally prioritised efficacy outcomes at the individual level. For example, pharmacokinetics and pharmacodynamics (PK/PD) modelling support optimisation of drug availability and duration to improve drug efficacy for a particular disease target within the treated individual. In contrast, prevention targets for infectious diseases require considering both the individual-level benefit and an understanding of a product’s public health benefit, including changes to immunity acquisition by delaying infections or via any impact on population-level transmission dynamics. This is essential when we consider medical interventions that impact susceptibility to infection or aim to prevent disease progression to clinically relevant presentations or severe disease.

While clinical trials evaluate the individual-level benefit of products, they do not estimate the total public health benefit over extended time frames or capture any potential benefits of interrupting onward transmission with population-level interventions. Modelling captures these dynamics to link individual and community benefits, allowing clinical trial evidence to be integrated into updated models or public health estimates, and can also support translating clinical trial results into implementation considerations. In early product development stages, population-level modelling can provide quantitative evidence linking an intervention’s minimum key performance criteria, such as efficacy or duration with its projected public health impact and benefit to communities towards meeting health targets, thus contributing to a robust evidence base^8^. While clinical evidence is essential to inform efficacy estimates and eventual registration and funding decisions for products, clinical evidence also informs model parameters, and modelling evidence in turn can improve clinical trial planning and design. For modelling evidence to be integrated within this process, it is essential for experts to be consulted early in the process to design research questions that modelling can address; an iterative loop between clinical trials, modelling, and expert opinion will reshape and transform criteria in guidance documents to support candidate selection.

Developing an accelerated and iterative process for the development of novel malaria prevention interventions should account for the following components.

### Identify unmet needs

The first step requires identifying who and where a malaria prevention intervention can meet the health needs of exposed populations. One approach is to consider the WHO-defined high burden high impact (HBHI) settings, ranking communities with mapped under-five mortality as a proxy for need. However, unmet needs can also be defined in communities without current prevention interventions, such as East Africa where SMC is not recommended or settings with perennial malaria transmission, or infants and pregnant women who are more vulnerable to severe outcomes and require higher consideration of drug safety^9^. Addressing health inequity, in particular low access to health services and first line treatment for symptomatic children and low bed net distribution coverage is essential to bridging the gaps of malaria programs and elimination strategies. As social, financial, and epidemiological factors drive change over time, including inequity, unmet needs should be revisited in an iterative manner to anticipate future scenarios. Defining these unmet needs will allow for prioritisation and evaluation of supply requirements for various timeframes and public health goals, such as for elimination.

### Define priority use-cases and potential intervention profiles

Once unmet needs have been articulated, the use-case definition is an important driver to prioritise development of a new intervention and should be defined rigorously at each TCP, iTPP, or PPC update to ensure candidates are addressing unmet health needs. A use-case defines the target population(s) who will receive a new malaria prevention intervention (children, pregnant women, all at-risk during an outbreak or humanitarian crisis, travellers to endemic regions), and where and when it should be deployed, ultimately influencing other criteria, including: product properties (mode-of-action, formulation, co-administration), logistic requirements (supply, delivery channel, cost-of-goods), intervention characteristics (protective efficacy, duration of protection, safety), and deployment strategy (deployment coverage, number of rounds). Considering these factors, modelling can evaluate how use-cases influence an intervention’s minimum criteria and vice versa.

### Define priority questions through community and expert engagement

In the early stages, shaping novel malaria intervention characteristics in guidance documents should also be guided by community engagement through discussions between global funders, health agencies, researchers, evidence generators (clinical trial investigators and modellers), product developers, local community leaders, and implementation specialists. These discussions should inform the identification of knowledge gaps, potential bottlenecks, and potential additional use-cases to refine the public health value proposition. Developing priority questions that inform modelling scenarios, such as comparing impact in seasonal and perennial settings or optimised number of rounds and timing of deployment, help guide evidence generation by informing clinical trial design and site selection targeting specific epidemiological settings, and the selection of model scenarios for predicting public health impact.

### Integrate clinical evidence

Pre-clinical and early clinical evidence of first candidates provides initial profiling of feasibility, safety, and PK/PD relationships. This then informs where, and for which use-cases, a candidate can meet health needs, the expectations of regulatory approval, and implementation factors such as supply, delivery, and deployment strategies. In addition, early clinical evidence for these candidates sets threshold criteria for selection of future candidates that are expected to achieve non-inferiority, and informs model parameterisation to narrow down the exploration space between individual-level effects and population-level impact.

### Integrate and refine modelling evidence

Mathematical models of malaria transmission can guide thinking along the entire product development pipeline from clinical trial translation to use-case decisions for implementation. Modelling can initially explore the major drivers of an intervention’s impact across a broad spectrum of scenarios to identify what clinical evidence needs to be generated early. In addition, models can support the clinical evidence generation process by identifying minimum thresholds, linking intervention characteristics to health goals to inform guidance documents to the level of detail required for appropriate candidate selection by product developers. Modelling can provide evidence on where potential candidates would be best implemented to maximise impact and help drive policy and procurement decisions.

### Living documents through process reiteration

Modelling can be integrated at each step in the development process to support articulating unmet needs, testing use-cases, translating clinical evidence, and setting achievable health goals to update guidance documents and adapt to new clinical evidence. As a result, evidence-based guidance and decision-making becomes an iterative process where modelling continuously supports the refinement of candidate profiles, rather than a linear process of development.

## Building a collaborative framework in practice

The Swiss Tropical and Public Health Institute Disease Modelling group and the Bill & Melinda Gates Foundation have developed a collaborative framework that integrates the process described above to iteratively inform next-generation iTPPs for seasonal, multi-seasonal, and second-generation SMC interventions for malaria prevention. In June 2021, workshops were organised to identify challenges and priority questions and to launch the platform with a broad range of experts and stakeholders, including those based in malaria endemic regions and ensuring gender equity. These discussions focused on the importance of guidance documents during R&D, patient-centred development, community advocacy for target population acceptability, and, when to stop or to continue funding new interventions. Working groups identified the following priorities for modelling: (1) describing trade-offs between intervention and implementation factors; (2) translating clinical trial and modelling evidence to inform policy and investments; (3) informing clinical trial design and identification of standard of care (SOC) comparators; (4) accounting for financial resources and cost-of-goods; and (5) defining burden reduction criteria and timelines for achieving elimination.

The collaborative framework generates modelling evidence using dynamic, individual-based malaria transmission models, such as the OpenMalaria model that was developed over a period of 15 years. Detailed models are coupled with additional analytical and statistical approaches to enable rapid and computationally efficient searches of multi-dimensional parameter spaces spanning a wide range of intervention characteristics and settings^8^. Modelling the mechanisms of individual-level factors and population-level transmission dynamics links predictions of public health burden reduction to key intervention characteristics. By predicting across an entire parameter space of intervention characteristics (for example, ranges for initial efficacy, duration of protection, and deployment coverage), modelling can quantify the importance and level of contribution of each characteristic to health outcomes and identify the minimum value at which intervention characteristics achieve defined health targets to directly inform iTPP criteria for different use-cases. Together with stakeholders from the 2021 convening, priority questions for modelling were ranked by most relevant scenarios to model at a first iteration for each given intervention by accounting for unmet health needs and priority use-cases. Clinical evidence, where and when available, informed parameter ranges to explore trade-offs and minimum criteria comprehensively. We give examples of this process in Table 1.

**Table 1:** Knowledge gaps addressed by integrating modelling at each development stage.

	Knowledge gaps	Modelling evidence
Clinical trial translation, planning, and design	• Which intervention properties drive effectiveness? • When and what type of evidence to generate?	• Key drivers of impact and early clinical data needed • Parameter range validation and translation to other use-cases • Candidate selection criteria
Use-case, target population, and setting	• Which use-cases to prioritize given unmet needs and intervention characteristics? • Which use-cases achieve the highest impact?	• Impact and minimum criteria requirements across a range of settings and age groups • Account for clinical evidence to re-evaluate use-cases
Deployment factors	• How can deployment frequency and timing be optimized? • What is the impact of mixed and layered intervention strategies?	• Scenarios of different strategies across use-cases to identify minimum criteria requirements that optimize effectiveness
Public health impact	• Does the novel intervention improve the standard of care? • Is the intervention cost-effective?	• Endpoint translation • Direct comparison of different interventions and evaluation periods

Knowledge gaps that can be addressed by population-level transmission modelling for next-generation interventions at different stages of development: before, during, and after early clinical trial studies to inform evidence generation and on-board data to better inform guidance documents with model-informed intervention minimum criteria; evaluating and re-evaluating use-cases iteratively to ensure intervention characteristics are appropriate for different use-cases to ensure high public health impact that addresses unmet health need; considering how deployment strategies and mixed interventions can be optimised to achieve health goals informed by modelling evidence; and directly generating predicted public health impact effectiveness for implementation given intervention efficacy characteristics to explore a large spectrum of possible scenarios and inform decision-making.

Models provide supportive information for decision-making or informing candidate selection, funding, and target product profiles alongside other diverse sources, including clinical evidence and expert opinion. Although not the sole source informing selection criteria, models have a unique role to play before and during clinical trials and as clinical evidence is being acquired. While the additional role of modelling is clear, all models have limitations. To be informative for selection criteria, models must capture essential disease dynamics while remaining simple enough to run. Population model prediction uncertainty comes from model structure and parameter values. For example, assumptions about human behaviour patterns or intervention effect will impact predictions. Some use cases, such as pregnant women or travellers, are more challenging to model due to limited data. Discussions around a model’s limitations in generating evidence to inform TPPs is inherently part of the process itself, which requires stakeholders to formulate specific questions that are fit for purpose. The conversations around models, their limitations, and sources of uncertainty, and how to translate predictions to tangible evidence become the most valuable output.

## Modelling to support clinical trial translation

Population-level transmission modelling can translate clinical efficacy outcomes to population-level effectiveness by integrating pharmacological evidence informing individual-level protection. Take, for example, monoclonal antibodies for infection prevention in seasonal settings. Currently very little is known about the PK/PD relationship of monoclonal antibodies. Previous modelling has shown that the protection of an anti-infective malaria monoclonal over time, informed by PK/PD data and models, is an important driver of public health impact^10^. Unlike vaccines, where immune correlates of protection are challenging to define, mAbs offer the potential to provide early PK/PD evidence. While the first-generation candidate CIS43LS has demonstrated high and prolonged protection, sufficient PD evidence from dose de-escalation data are not yet available^11^. Modelling can address this knowledge gap by generating a comprehensive spectrum of PD characteristics from forthcoming early clinical trials to provide a broad range of potential impact predictions that can be refined iteratively as more data is generated.

## Modelling to interpret and define public health impact targets required to guide intervention development

For malaria prevention, imperfect tools, such as perennial malaria chemoprevention (PMC), SMC, and RTS,S vaccination, have been deployed in moderate to high transmission settings. The public health and cost-effectiveness targets for novel interventions can be informed by the standard-of-care (SOC) comparators when the use-case is well defined and alternative interventions already exist. In the absence of a comparator, ranges of desired health targets can be iteratively assessed, but comparison of effectiveness to existing prevention technologies is critical to refine the value proposition for further R&D investment. Without population-based modelling before Phase 3 clinical trials, it is challenging to evaluate the effectiveness of candidates with early clinical data alone. Modelling can quantify effectiveness for different intervention characteristics, use-cases, and evaluation periods, as well as easily translate different endpoints to guide policy decisions. For example, the public health impact and cost-effectiveness of the RTS,S malaria vaccine was evaluated by four independent modelling groups using different mathematical models of malaria transmission. This body of work estimated disability-adjusted life years (DALYs) for both a three-dose and four-dose vaccine schedule more than four years prior to the start of the pilot studies. These cost-effectiveness estimates demonstrated that a partially efficacious vaccine could have significant impact at the population level, critical for the WHO’s recent recommendation of RTS,S^12,13^.

## Modelling to iteratively update guidance documents and readdress use-cases

Guidance documents for mAbs, next-generation chemoprevention, and vaccines in development currently prioritise preventing clinical illness and severe disease in vulnerable populations, including infants, children under five, and pregnant women. When use-cases are uncertain, modelling can simulate scenarios for different age groups, seasonal profiles, and transmission intensities to demonstrate where interventions will be most impactful and reassess when new evidence is onboarded. For example, WHO recommends malaria vaccines for children aged five to 17 months old in perennial settings with potential for seasonal application in children under five years old. However, these target ages and seasonal use-case only came to light as clinical evidence was accrued because the vaccine prevented infections for a shorter duration than hoped during the Phase 3 studies^12,14^. Currently, the efficacy profiles of first-generation anti-infective vaccines like RTS,S and, potentially, R21 support their use in seasonal settings. Yet, many questions remain regarding implementation factors, combinations of the vaccine with oral chemoprevention programs, and impact across different epidemiological settings. Modelling can support this by predicting the likely impact of such factors prior to expensive clinical trials and pilot studies and explore the feasibility of these tools to achieve future elimination targets.

The malaria vaccine example described here provides a lesson to heed for novel malaria intervention development. Firstly, use-cases of novel interventions should be re-evaluated with an improved understanding of the limits of an intervention’s efficacy and duration. Thus, understanding the efficacy and duration of novel malaria interventions as early as possible will help not only to select or reject candidates and optimise duration properties, but also assess and reassess appropriate use-cases iteratively. Modelling supports this by demonstrating the trade-offs between deployment factors and intervention characteristics to identify minimum criteria well before Phase 3 trials. In some cases it will provide efficacy and duration cut-offs (clear ‘no-go’ criteria) for candidate use in different uses-cases. However, if early clinical evidence suggests candidates will struggle to ever meet these initial efficacy and duration requirements defined by iTPPs use-cases, the community should not always discard classes of candidates or novel interventions, but critically and discriminately revaluate if there is value of this efficacy and duration profile for another use-case for malaria prevention.

## Conclusion

With increasing threats of malaria drug and insecticide resistance, there is now a need for novel malaria prevention interventions to improve public health impact. As the malaria community moves forward to invest in next-generation interventions, guidance documents are crucial to ensure that the best evidence supports the criteria for candidate section and decision-making for implementation. While clinical evidence and expert opinion will initially play an essential role in informing these criteria, mathematical modelling can accelerate this process and provide robust evidence of candidate characteristics and deployment strategies that are likely to lead to a higher public health impact for different use-cases and enhance the value proposition for a given development candidate. Modelling should be incorporated early in the evidence generation process, to improve the translation of clinical trial efficacy estimates and to support use-case and implementation strategy decisions. Public health impact predictions from modelling studies that include detailed intervention dynamics are currently underutilised for setting selection criteria early in development. Thus, including such analytic tools early on provides a unique opportunity to accelerate the development of malaria interventions for optimised use-cases and deployment strategies.
